# Phase II trial of humanized anti-Lewis Y monoclonal antibody for advanced hormone receptor-positive breast cancer that progressed following endocrine therapy

**DOI:** 10.6061/clinics/2021/e3146

**Published:** 2021-09-28

**Authors:** Laura Testa, Max Mano, Roberto Jun Arai, Renata Colombo Bonadio, Sergio V. Serrano, Marina M Costa Zorzetto, Susanne Crocamo, Oren Smaletz, Ruffo Freitas-Junior, Paulo M. Hoff

**Affiliations:** I Instituto do Cancer do Estado de Sao Paulo (ICESP), Hospital das Clinicas HCFMUSP, Faculdade de Medicina, Universidade de Sao Paulo, Sao Paulo, SP, BR.; II Hospital de Cancer de Barretos, Fundaçao Pio XII, Barretos, SP, BR.; III Instituto Nacional do Cancer - INCA, Rio de Janeiro, RJ, BR.; IV Recepta Biopharma e Hospital Israelita Albert Einstein, Sao Paulo, SP, BR.; V Universidade Federal de Goias, Goiania, GO, BR.; VI Oncologia D’or, Sao Paulo, SP, BR.

**Keywords:** Breast Cancer, Metastatic, Anti-Lewis Y, Monoclonal Antibody, Targeted Therapy

## Abstract

**OBJECTIVES:**

The Lewis-Y antigen is expressed in 44%-90% of breast cancers (BCs). The expression of the antigen in carcinoma tissue differs from that in normal tissues. This study aimed to evaluate the clinical benefit of the humanized anti-Lewis Y monoclonal antibody, hu3S193, in advanced hormone receptor-positive and Lewis Y-positive BC after administration of endocrine therapy (ET).

**METHODS:**

A single-arm phase II study was conducted in seven centers. Patients with advanced hormone receptor-positive BC who failed first-line ET were included. The inclusion criterion was the observation of tumoral expression of the Lewis Y antigen during immunohistochemistry. The treatment comprised hu3S193 antibody administration at weekly intravenous doses of 20 mg/m^2^ for 8-week cycles. The primary endpoint was the clinical benefit rate. ClinicalTrials.gov NCT01370239.

**RESULTS:**

The study stopped accrual following an unplanned interim analysis as the hu3S193 antibody lacked sufficient activity to justify continuation of the study. Twenty-two patients were enrolled, of whom 21 were included in the efficacy analysis. The clinical benefit rate was 19%, with four patients presenting with stable disease after 24 weeks. One patient with prolonged stable disease received medication for over 2 years. No partial or complete responses were observed. The median time to progression and overall survival was 5.4 and 37.5 months, respectively.

**CONCLUSIONS:**

The humanized anti-Lewis Y monoclonal antibody, hu3S193, exhibited insufficient activity in this cohort. However, the possibility of activity in a more strictly selected subgroup of patients with higher levels of Lewis Y tumoral expression cannot be overlooked.

## INTRODUCTION

Breast cancer (BC) is the most common cancer and leading cause of cancer-related deaths among women (1). The disease has various subtypes with differing prognostic and therapeutic implications. Estrogen receptor expression occurs in 65% of invasive ductal carcinoma cases and most lobular carcinoma cases (2). Endocrine therapy (ET) is one of the standard first-line treatments for advanced hormone receptor-positive BC (3). ET has improved recently following the incorporation of targeted therapies, such as cyclin-dependent kinase (CDK) and mTOR inhibitors (4-10); however, these treatment strategies are not yet widely available in the public health system.

After first-line treatment failure, the response to subsequent lines of ET or cytotoxic chemotherapy decreased dramatically. Treatment with second-line ET alone is associated with a median progression-free survival of only 3-5 months, with response and clinical benefit rates in the range of 20%-30% and 30%-40%, respectively (11-13). This highlights the importance of incorporating new treatment strategies in clinical practice and studying novel targets.

Alterations in blood-related antigens are often associated with neoplastic transformation (14). The Lewis-Y antigen is a member of a family of blood-related antigens, and its expression is restricted to granulocytes and epithelial surfaces in adults (15). It is expressed on the surface of 60%-90% of carcinomas (16). The Lewis-Y antigen is expressed in 44%-90% of BC cases, with variations occurring depending on the methodology used (14,17,18). In addition, its expression in cases of BC is correlated with a worse prognosis and a more advanced disease stage (14,19).

Although the Lewis-Y antigen is expressed in both normal and neoplastic cells, its expression distribution differs between the two tissue types. Expression in normal epithelial tissue is restricted to the secretory borders of epithelial surfaces, making it less accessible to the circulation. In contrast, the expression of the antigen is very high on all surfaces of carcinoma cells, including luminal surfaces. This differentiated expression pattern makes it an attractive target for treatment with monoclonal antibodies. This hypothesis is reinforced by the results of previous preclinical studies, which have demonstrated the preferential localization of anti-Lewis Y antibodies in tumor tissue after its injection into the circulation of experimental animals (20-22).

The murine monoclonal antibody 3S193 was developed to recognize the Lewis-Y antigen of the MCF-7 breast carcinoma cell line following consideration of these studies’ data. The murine 3S193 antibody was specific to the Lewis-Y antigen and reactive against cells expressing the antigen (23). Therefore, a humanized immunoglobulin G monoclonal antibody, hu3S193, was developed for in-human studies (16). The safety of the hu3S193 antibody and preliminary evidence of its activity in several tumor types, including BC, have been demonstrated previously (24,25).

The objective of this study was to evaluate the efficacy of the hu3S193 monoclonal antibody in patients with advanced hormone receptor-positive BC after prior ET administration.

## PATIENTS AND METHODS

### Ethical approval and consent to participate

Ethical approval was obtained from the ethics committee of each participating institution. All participants provided informed consent to participate in this study.

### Patients

Patients with hormone receptor-positive metastatic or locally advanced BC not amenable to curative treatment and tumoral expression of the estrogen or progesterone receptor (or both) according to immunohistochemistry (IHC) results were included in this study. Patients should have progressed following one or more lines of ET, including adjuvant ET. Patients who underwent prior treatment with up to one line of chemotherapy for metastatic disease could be included. This study was previously presented at the 2017 San Antonio Breast Cancer Symposium, San Antonio, USA and 2018 Brazilian Breast Cancer Symposium, Pirenópolis, Brazil.

IHC revealed that all patients exhibited tumor expression of the Lewis-Y antigen. Tumor expression of the Lewis Y antigen by IHC was performed by central analysis (LIM 14 of the Medical School of the University of São Paulo). The samples were considered positive if any reaction was observed in the membrane of the tumor cells.

Other inclusion criteria included an Eastern Cooperative Oncology Group performance status score of 0 or 1 and preserved organic functions.

The exclusion criteria included overexpression of human epidermal growth factor receptor 2 (defined as IHC 3+ or positive fluorescence *in situ* hybridization), life-threatening visceral metastatic disease (defined as extensive hepatic involvement, symptomatic pulmonary lymphangitic carcinomatosis, and cerebral or leptomeningeal metastases), and the need for systemic corticosteroid or immunosuppressive agent administration.

### Outcomes

The primary endpoint was the clinical benefit rate of the hu3S193 monoclonal antibody, defined as complete or partial response, or stable disease for at least 24 weeks.

The secondary endpoints were the following: response and non-progression rate, overall survival, time to progression, and safety. The response rate was defined as the proportion of patients who presented with a complete or partial response. The non-progression rate was defined as the proportion of patients who presented with complete or partial response or stable disease, regardless of the duration of the latter.

Overall survival was defined as the time from the first study drug dose until death from any cause. Patients without this event were censored on the date of the last follow-up. Time to progression was defined as the time from the first study drug dose to any clinical or radiological progression. Patients who did not experience these events were censored at the last follow-up date or the date of death without progression.

### Study design

The HumanaH trial was a phase II single-arm trial, which was coordinated by the Instituto do Câncer do Estado de São Paulo (NCT01370239) and conducted in seven centers in Brazil.

The treatment comprised weekly administration of the hu3S193 antibody with intravenous infusions of 20 mg/m^2^. Each treatment cycle lasted for 8 weeks. Treatment continued until clinical or radiological disease progression, unacceptable toxicity, withdrawal of consent, or a decision by the investigator. The protocol allowed for dose reduction. In cases of grade 3 or 4 non-allergic toxicities, treatment would have been delayed for up to 14 days until the toxicities were reduced to grade 1 or 2 and resumed with a 25% dose reduction. Treatment would have been discontinued in cases where no improvement occurred within 14 days.

### Study supervision

The Ethics Committee of each participating institution approved the study, which was conducted in accordance with the principles of the Declaration of Helsinki and International Conference on Harmonization Good Clinical Practice guidelines. The patients read and signed an informed consent form before undergoing any of the study procedures.

Recepta Biopharma (S. Paulo, Brazil) was granted the use of hu3S193 antibodies. The study was sponsored by the Conselho Nacional de Desenvolvimento Científico e Tecnológico (CNPq), Grant N^0^ 52/2009 edital CNPq.

The authors were responsible for the design of the study, analyzing the results, and writing of the manuscript.

### Assessments

Radiological responses were evaluated every 8 weeks. Mandatory radiological images comprised computed tomography or magnetic resonance imaging of the thorax, abdomen, and pelvis. Brain imaging and bone scans were performed if clinically indicated. The radiological responses were evaluated based on the Response Evaluation Criteria in Solid Tumors version 1.1.

Adverse events were monitored, and their severity was scaled using the National Cancer Institute Common Terminology Criteria for Adverse Events version 4.03.

Human anti-human antibodies (HAHA) tests were performed periodically at baseline, week 8 of each cycle, at the end of treatment, and if immune-mediated adverse events occurred. Samples were analyzed using enzyme-linked immunosorbent assay. Treatment with hu3S193 antibodies should be discontinued if the patient tests positive for HAHA.

### Statistical analyses

Efficacy analyses were performed according to the intention-to-treat principle. All patients receiving at least one dose of hu3S193 were included in the safety analysis.

We calculated our sample size based on the historical clinical benefit rates of three studies that included a similar profile of patients treated with ET (11,12,26). A historical and clinical benefit rate of hu3S193 of 40% and 60%, respectively, was estimated. A total of 60 patients would be required if a two-sided alpha error of 5%, power of 85%, and withdrawal rate of 10% occur.

Because many patients discontinued the study because of disease progression, an unplanned interim analysis was performed. The interim analysis detected a two-sided alpha error of 5%, a beta error of 20%, and a historical and clinical benefit rate with hy3S193 of 40% and 60%, respectively. Trials should be terminated if 10 or fewer patients responded after 21 patients were tested at the first stage, according to Simon’s two-stage design. If 11 or more patients respond, the trial should continue to the second stage to include a total of 60 patients.

Descriptive statistics were used to summarize patient characteristics, radiological responses, and adverse events. Continuous variables are presented as medians and ranges, while categorical variables are presented as absolute and relative frequencies. Clinical benefit and non-progression rates were presented as proportions and 95% confidence intervals (CIs). Survival analyses were performed using the Kaplan-Meier method. Stata software version 14 (StataCorp, Texas, USA) was used to perform statistical analyses.

## RESULTS

### Patients characteristics

Forty-nine patients were screened for inclusion in the trial between November 2013 and July 2015, of whom 23 were initially considered eligible. One eligible patient was lost to follow-up before treatment initiation. Twenty-two patients received at least one dose of hu3S193 and were included in the safety analyses.

**Figure 1:**
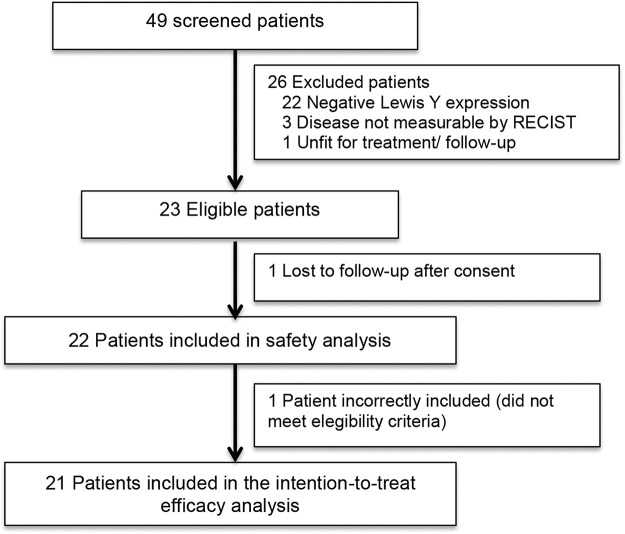
CONSORT diagram. RECIST, Response Evaluation Criteria in Solid Tumors.

One of these patients was incorrectly included in the trial, as she had lung metastasis which was mistaken for interstitial lung disease. This patient was not included in the efficacy analysis because she did not meet the study eligibility criteria. The remaining 21 patients were included in the efficacy analyses. The CONSORT diagram is shown in Figure 1.

The median age was 54 years (range, 39-79 years). All patients were estrogen receptor-positive, and 17 (77.3%) had progesterone receptor-positive BC. Sixteen patients (72.7%) had received chemotherapy previously. The patient characteristics are summarized in Table 1.

### Efficacy

The study was discontinued after an unplanned interim analysis because of futility. The hu3S193 antibody did not exhibit sufficient activity to justify the continuation of the study.

**Figure 2:**
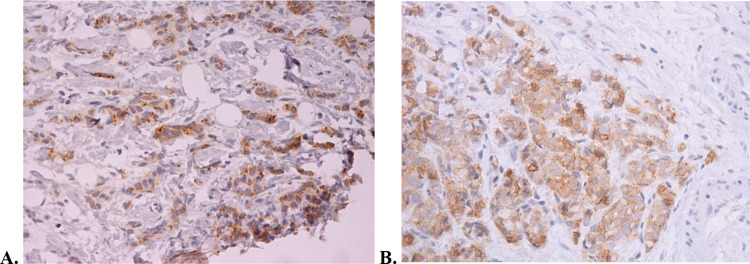
Lewis Y antigen expression of the patient with stable disease for more than 2 years who was administered hu3S193 according to immunohistochemistry. A. Predominant membrane Lewis Y staining ×200. B. Membrane and cytoplasm Lewis Y staining ×200.

The median follow-up period was 19 months at the time of study interruption. Four patients experienced clinical benefits because of hu3S193 antibody administration, corresponding to a clinical benefit rate of 19% (95% CI, 2.5%-35.8%). All patients who experienced clinical benefits presented with stable disease for at least 24 weeks. One patient received medication for more than 2 years and had stable disease. This patient presented with elevated Lewis-Y antigen expression, as shown in Figure 2. None of the patients exhibited partial or complete responses. The non-progression rate was 57.1% (95% CI, 36%-78.3%). The radiological responses are summarized in Table 2. The median time to progression was 5.4 months (95% CI, 1.7-18.9 months).

**Figure 3:**
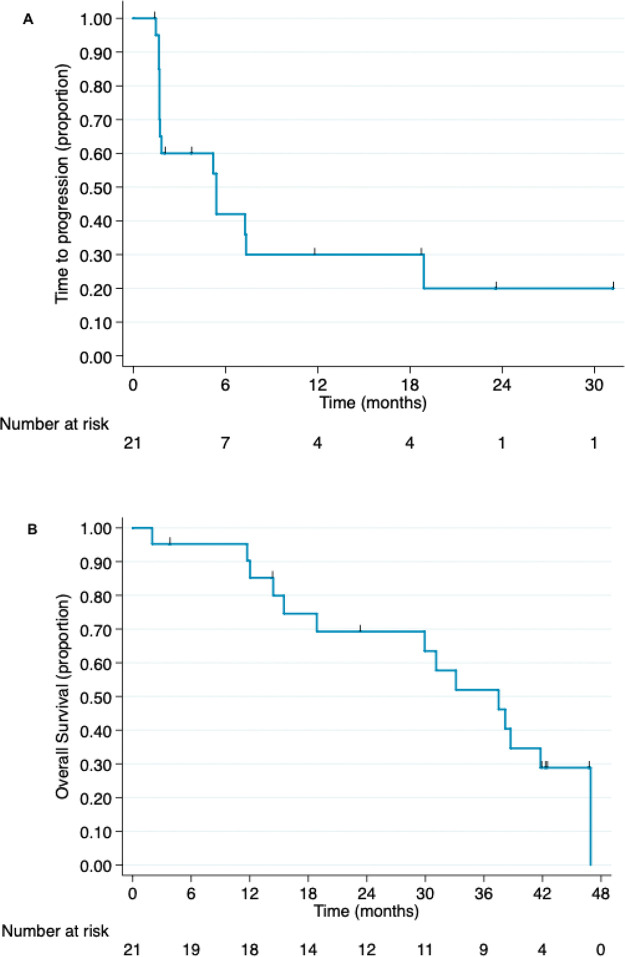
Kaplan-Meier curves of (A) time to progression and (B) overall survival for patients with advanced hormone-receptor positive breast cancer who were administered anti-Lewis Y monoclonal antibody (hu3S193) (intention-to-treat population).

The overall survival data were updated following a median follow-up of 31.1 months. Fourteen patients died during the study period. The median overall survival was 37.5 months (95% CI, 15.5 months-not reached). The time to progression and overall survival curves are presented in Figure 3.

### Adverse events

Two patients experienced serious adverse events potentially related to the study treatment. One patient presented with dyspnea, and the other experienced vomiting, dyspnea, and infection (two episodes of pneumonia and one instance of urinary tract infection). Both patients recovered fully.

All patients who received at least one dose of treatment experienced non-serious adverse events potentially related to the study treatment. The most common of these were headache (n=11), cough (n=10), nausea (n=7), vomiting (n=7), and musculoskeletal pain (n=6). Major events (grade 3 or 4) were uncommon. Adverse events are shown in Table 3 and ordered according to grade. 

No deaths related to study treatment occurred.

## DISCUSSION

In this phase II study, patients with advanced hormone receptor-positive BC that progressed after ET was administered underwent treatment with a humanized antibody that targeted the Lewis-Y antigen, hu3S193. This agent did not exhibit sufficient activity to justify the continuation of the study, which was concluded following an unplanned interim analysis because of futility. Among 21 patients, only 4 had stable disease after 24 weeks and none had partial or complete responses (clinical benefit rate of 19%).

Another phase II study evaluating the efficacy of hu3S193 in patients with advanced, platinum-resistant, ovarian, tubal, or primary peritoneal cancer had similar results (27). This study was also interrupted prematurely because of low drug activity and had a clinical benefit rate of 23% (stable disease confirmed at 24 weeks) with no documented partial or complete responses.

However, one patient experienced prolonged clinical benefits following treatment and attained stable disease after 2 years of hu3S193 antibody treatment. The patient exhibited elevated Lewis-Y tumor expression. Thus, a more strictly selected patient population (characterized by elevated Lewis-Y tumoral expression) may still benefit from the treatment.

Treatment with the hu3S193 antibody proved to be safe and well-tolerated. The adverse events potentially related to the study treatment were mostly low-grade and manageable, consisting mainly of headaches, cough, musculoskeletal pain, nausea, and vomiting. No serious adverse events mediated by the immune system were observed.

It is important to note that significant changes in the treatment of hormone receptor-positive BC occurred after the conclusion of this study because of the advent of CDK inhibitors, as mentioned previously. The benefit of combining these drugs with ET has been demonstrated in both first-line and second-line settings (4-9). Three drugs of this class (palbociclib, ribociclib, and abemaciclib) have changed the treatment algorithm for advanced or metastatic estrogen receptor-positive BC and have been incorporated into clinical practice in many countries following robust improvements in progression-free survival rates (28).

In conclusion, the anti-Lewis Y antibody, hu3S193, exhibited insufficient efficacy in patients with advanced hormone receptor-positive and Lewis-Y antigen-positive BC that progressed after ET was administered. Its potential efficacy in patients with increased Lewis-Y antigen tumoral expression cannot be ruled out. No relevant toxicity with hu3s193 was observed.

## AUTHOR CONTRIBUTIONS

Testa L, Mano M, Arai RJ, Smaletz O and Hoff PM were responsible for the concept and design. Testa L, Mano M, Serrano SV, Zorzetto MMC, Crocamo S and Freitas-Junior R were responsible for the patient recruitment. Testa L, Mano M, Arai RJ, Bonadio RC and Hoff PM were responsible for the data acquisition and analysis. Testa L, Mano M, Arai RJ, Bonadio RC and Freitas-Junior R were responsible for the manuscript writing. All of the authors read and approved the final version of the manuscript.

## Figures and Tables

**Table 1:** Patients characteristics.

	No.	%
Population size	22	
Median age (range), years	54 (39-79)	
Race		
White	15	68.2
Black or mulatto	6	27.3
Asian	1	4.5
Estrogen receptor		
Positive	22	100
Negative	0	0
Progesterone receptor		
Positive	17	77.3
Negative	5	22.7
Metastasis sites		
Bone	13	59
Lung	8	36.3
Liver	4	18.2
Lymph nodes	2	9.1
Pleura	1	4.5
Soft tissue	1	4.5
Previous surgery	18	81.8
Endocrine therapy	22	100
Adjuvant	18	81.8
Palliative	13	59
Chemotherapy	16	72.7
Neoadjuvant or adjuvant	15	68.2
Palliative	1	4.5
Adjuvant radiotherapy	14	63.3

Abbreviations: No., number.

**Table 2:** Radiological response in the intention-to-treat population with advanced hormone-receptor positive breast cancer treated with anti-Lewis Y monoclonal antibody (hu3S193).

	No. of patients (N=21)	%
Complete response	0	0
Partial response	0	0
Stable disease (SD)		
SD as best radiological response	12	57.1%
SD confirmed after 24 weeks	4	19%
Disease progression (as best radiological response)	8	38.1%
Not evaluable*	1	4.8%

Abbreviations: No., number; SD, stable disease.

* One patient presented with clinical worsening in the first weeks of treatment and did not undergo imaging evaluation after treatment.

**Table 3:** Adverse events classified according to grade in patients with advanced hormone-receptor positive breast cancer treated with anti-Lewis Y monoclonal antibody (hu3S193).

Adverse events	No. of patients (%) (N=22)			
	Grade			
	1	2	3	4
Hematologic				
Anemia	0 (0)	3 (13.6)	0 (0)	0 (0)
Neutropenia	0 (0)	0 (0)	1 (4.5)	0 (0)
Thrombocytopenia	1 (4.5)	1 (4.5)	0 (0)	0 (0)
Biochemistry tests				
Alanine aminotransferase elevation	1 (4.5)	1 (4.5)	0 (0)	0 (0)
Aspartate aminotransferase elevation	2 (9.1)	0 (0)	1 (4.5)	0 (0)
Hypercalcemia	1 (4.5)	1 (4.5)	0 (0)	0 (0)
Gastrointestinal				
Abdominal discomfort	1 (4.5)	1 (4.5)	0 (0)	0 (0)
Constipation	3 (13.6)	1 (4.5)	0 (0)	0 (0)
Diarrhea	5 (22.7)	0 (0)	0 (0)	0 (0)
Nausea	5 (22.7)	4 (18.2)	0 (0)	0 (0)
Vomiting	7 (31.8)	1 (4.5)	0 (0)	0 (0)
Other				
Musculoskeletal pain	6 (27.3)	2 (9.1)	0 (0)	0 (0)
Cough	8 (36.4)	4 (18.2)	0 (0)	0 (0)
Fatigue	3 (13.6)	2 (9.1)	1 (4.5)	0 (0)
Dizziness	5 (22.7)	1 (4.5)	0 (0)	0 (0)
Headache	9 (40.9)	5 (22.7)	0 (0)	0 (0)
Tremor	2 (9.1)	1 (4.5)	0 (0)	0 (0)
Itchiness	4 (18.2)	0 (0)	0 (0)	0 (0)
Skin rash	1 (4.5)	2 (9.1)	0 (0)	0 (0)

## References

[1] Bray F, Ferlay J, Soerjomataram I, Siegel RL, Torre LA, Jemal A (2018). Global cancer statistics 2018: GLOBOCAN estimates of incidence and mortality worldwide for 36 cancers in 185 countries. CA Cancer J Clin.

[2] Luveta J, Parks RM, Heery DM, Cheung KL, Johnston SJ (2020). Invasive Lobular Breast Cancer as a Distinct Disease: Implications for Therapeutic Strategy. Oncol Ther.

[3] Telli ML, Gradishar WJ, Ward JH (2019). NCCN Guidelines Updates: Breast Cancer. J Natl Compr Canc Netw.

[4] Finn RS, Martin M, Rugo HS, Jones S, Im SA, Gelmon K (2016). Palbociclib and Letrozole in Advanced Breast Cancer. N Engl J Med.

[B05] Cristofanilli M, Turner NC, Bondarenko I, Ro J, Im SA, Masuda N (2016). Fulvestrant plus palbociclib versus fulvestrant plus placebo for treatment of hormone-receptor-positive, HER2-negative metastatic breast cancer that progressed on previous endocrine therapy (PALOMA-3): final analysis of the multicentre, double-blind, phase 3 randomised controlled trial. Lancet Oncol.

[B06] Hortobagyi GN, Stemmer SM, Burris HA, Yap YS, Sonke GS, Paluch-Shimon S (2016). Ribociclib as First-Line Therapy for HR-Positive, Advanced Breast Cancer. N Engl J Med.

[B07] Sledge GW, Toi M, Neven P, Sohn J, Inoue K, Pivot X (2017). MONARCH 2: Abemaciclib in Combination With Fulvestrant in Women With HR+/HER2- Advanced Breast Cancer Who Had Progressed While Receiving Endocrine Therapy. J Clin Oncol.

[B08] Goetz MP, Toi M, Campone M, Sohn J, Paluch-Shimon S, Huober J (2017). MONARCH 3: Abemaciclib As Initial Therapy for Advanced Breast Cancer. J Clin Oncol.

[9] Slamon DJ, Neven P, Chia S, Fasching PA, De Laurentiis M, Im SA (2018). Phase III Randomized Study of Ribociclib and Fulvestrant in Hormone Receptor-Positive, Human Epidermal Growth Factor Receptor 2-Negative Advanced Breast Cancer: MONALEESA-3. J Clin Oncol.

[10] Baselga J, Campone M, Piccart M, Burris HA, Rugo HS, Sahmoud T (2012). Everolimus in postmenopausal hormone-receptor-positive advanced breast cancer. N Engl J Med.

[11] Howell A, Robertson JF, Quaresma Albano J, Aschermannova A, Mauriac L, Kleeberg UR (2002). Fulvestrant, formerly ICI 182,780, is as effective as anastrozole in postmenopausal women with advanced breast cancer progressing after prior endocrine treatment. J Clin Oncol.

[12] Osborne CK, Pippen J, Jones SE, Parker LM, Ellis M, Come S (2002). Double-blind, randomized trial comparing the efficacy and tolerability of fulvestrant versus anastrozole in postmenopausal women with advanced breast cancer progressing on prior endocrine therapy: results of a North American trial. J Clin Oncol.

[13] Iwamoto T, Fujisawa T, Shien T, Araki K, Sakamaki K, Sangai T (2020). The efficacy of sequential second-line endocrine therapies (ETs) in postmenopausal estrogen receptor-positive and HER2-negative metastatic breast cancer patients with lower sensitivity to initial ETs. Breast Cancer.

[14] Madjd Z, Parsons T, Watson NF, Spendlove I, Ellis I, Durrant LG (2005). High expression of Lewis y/b antigens is associated with decreased survival in lymph node negative breast carcinomas. Breast Cancer Res.

[15] Dettke M, Pálfi G, Loibner H (2000). Activation-dependent expression of the blood group-related lewis Y antigen on peripheral blood granulocytes. J Leukoc Biol.

[16] Scott AM, Geleick D, Rubira M, Clarke K, Nice EC, Smyth FE (2000). Construction, production, and characterization of humanized anti-Lewis Y monoclonal antibody 3S193 for targeted immunotherapy of solid tumors. Cancer Res.

[17] Hellström I, Garrigues HJ, Garrigues U, Hellström KE (1990). Highly tumor-reactive, internalizing, mouse monoclonal antibodies to Le(y)-related cell surface antigens. Cancer Res.

[18] Saleh MN, Sugarman S, Murray J, Ostroff JB, Healey D, Jones D (2000). Phase I trial of the anti-Lewis Y drug immunoconjugate BR96-doxorubicin in patients with lewis Y-expressing epithelial tumors. J Clin Oncol.

[19] Steplewska-Mazur K, Gabriel A, Zajecki W, Wylezoł M, Glück M (2000). Breast cancer progression and expression of blood group-related tumor-associated antigens. Hybridoma.

[20] Clarke K, Lee FT, Brechbiel MW, Smyth FE, Old LJ, Scott AM (2000). In vivo biodistribution of a humanized anti-Lewis Y monoclonal antibody (hu3S193) in MCF-7 xenografted BALB/c nude mice. Cancer Res.

[B21] Lövqvist A, Humm JL, Sheikh A, Finn RD, Koziorowski J, Ruan S (2001). PET imaging of (86)Y-labeled anti-Lewis Y monoclonal antibodies in a nude mouse model: comparison between (86)Y and (111)In radiolabels. J Nucl Med.

[22] Pai-Scherf LH, Carrasquillo JA, Paik C, Gansow O, Whatley M, Pearson D (2000). Imaging and phase I study of 111In- and 90Y-labeled anti-LewisY monoclonal antibody B3. Clin Cancer Res.

[23] Kitamura K, Stockert E, Garin-Chesa P, Welt S, Lloyd KO, Armour KL (1994). Specificity analysis of blood group Lewis-y (Le(y)) antibodies generatedagainst synthetic and natural Le(y) determinants. Proc Natl Acad Sci U S A.

[24] Scott AM, Tebbutt N, Lee FT, Cavicchiolo T, Liu Z, Gill S (2007). A phase I biodistribution and pharmacokinetic trial of humanized monoclonal antibody Hu3s193 in patients with advanced epithelial cancers that express the Lewis-Y antigen. Clin Cancer Res.

[25] Krug LM, Milton DT, Jungbluth AA, Chen LC, Quaia E, Pandit-Taskar N (2007). Targeting Lewis Y (Le(y)) in small cell lung cancer with a humanized monoclonal antibody, hu3S193: a pilot trial testing two dose levels. J Thorac Oncol.

[26] Chia S, Gradishar W, Mauriac L, Bines J, Amant F, Federico M (2008). Double-blind, randomized placebo controlled trial of fulvestrant compared with exemestane after prior nonsteroidal aromatase inhibitor therapy in postmenopausal women with hormone receptor-positive, advanced breast cancer: results from EFECT. J Clin Oncol.

[27] Smaletz O, Diz MD, do Carmo CC, Sabbaga J, Cunha-Junior GF, Azevedo SJ (2015). A phase II trial with anti-Lewis-Y monoclonal antibody (hu3S193) for the treatment of platinum resistant/refractory ovarian, fallopian tube and primary peritoneal carcinoma. Gynecol Oncol.

[28] Gao JJ, Cheng J, Bloomquist E, Sanchez J, Wedam SB, Singh H (2020). CDK4/6 inhibitor treatment for patients with hormone receptor-positive, HER2-negative, advanced or metastatic breast cancer: a US Food and Drug Administration pooled analysis. Lancet Oncol.

